# Supplementary material for: An ultra-short-acting benzodiazepine in thalamic nucleus reuniens undermines fear extinction via intermediation of hippocamposeptal circuits
Source: Commun Biol. 2024 Jun 14;7:728. doi: 10.1038/s42003-024-06417-w (PMC11178775; doi:10.1038/s42003-024-06417-w)
Supplement: Supplementary file 2 — Supplementary Information [file 42003_2024_6417_MOESM2_ESM.pdf]

Supplementary Information for

An ultra-short-acting benzodiazepine in thalamic nucleus reuniens undermines fear extinction via intermediation of hippocamposeptal circuits

Hoiyin Cheung[1–3,#], Tong-Zhou Yu[4,#], Xin Yi[2,4,#], Yan-Jiao Wu[2], Qi Wang[2], Xue Gu[2], Miao Xu[4], Meihua Cai[1], Wen Wen[1], Xin-Ni Li[4], Ying-Xiao Liu[4], Ying Sun[1], Jijian Zheng[1], Tian-Le Xu[1,2,5], Yan Luo[3,*], Ma-Zhong Zhang[1,*], Wei-Guang Li[2,4,6,*]

[1]Center for Brain Science, Department of Anesthesiology and Pediatric Clinical Pharmacology Laboratory, Shanghai Children's Medical Center, National Children's Medical Center, Shanghai Jiao Tong University School of Medicine, Shanghai 200127, China
[2]Department of Anatomy and Physiology, Shanghai Jiao Tong University School of Medicine, Shanghai 200025, China
[3]Department of Anesthesiology, Ruijin Hospital, Shanghai Jiao Tong University School of Medicine, Shanghai 200025, China
[4]Department of Rehabilitation Medicine, Huashan Hospital, Institute for Translational Brain Research, State Key Laboratory of Medical Neurobiology and Ministry of Education Frontiers Center for Brain Science, Fudan University, Shanghai 200032, China
[5]Songjiang Hospital and Songjiang Research Institute, Shanghai Jiao Tong University School of Medicine, Shanghai 201600, China
[6]Ministry of Education-Shanghai Key Laboratory for Children's Environmental Health, Xinhua Hospital, Shanghai Jiao Tong University School of Medicine, Shanghai200092, China
[#]These authors contributed equally

[*]Corresponding author:
Wei-Guang Li, PhD (E-mail: liwg@fudan.edu.cn)
Ma-Zhong Zhang, PhD (E-mail: zmzscmc@shsmu.edu.cn)
Yan Luo, PhD (E-mail: ly11087@rjh.com.cn)

Supplementary Information provided in this file:

Supplementary Fig. 1. Behavioral effects of remimazolam with different doses.
Supplementary Fig. 2. Effects of RE-specific infusion of remimazolam on locomotor activity and basal anxiety.
Supplementary Fig. 3. Flumazenil abolishes the effects of remimazolam on RE synaptic transmission and fear extinction behaviors.
Supplementary Fig. 4. Verification of Gabrg2 knock down in RE and related behavioral effects.
Supplementary Fig. 5. Effects of chemogenetic activating RE-vHPC projectors on the remimazolam responsiveness to fear extinction.
Supplementary Fig. 6. Effects of optogenetic activation of LS → RE projections on locomotor activity and basal anxiety.
Supplementary Fig. 7. Electrophysiological characterization of synaptic projections from the vHPC to the LS.

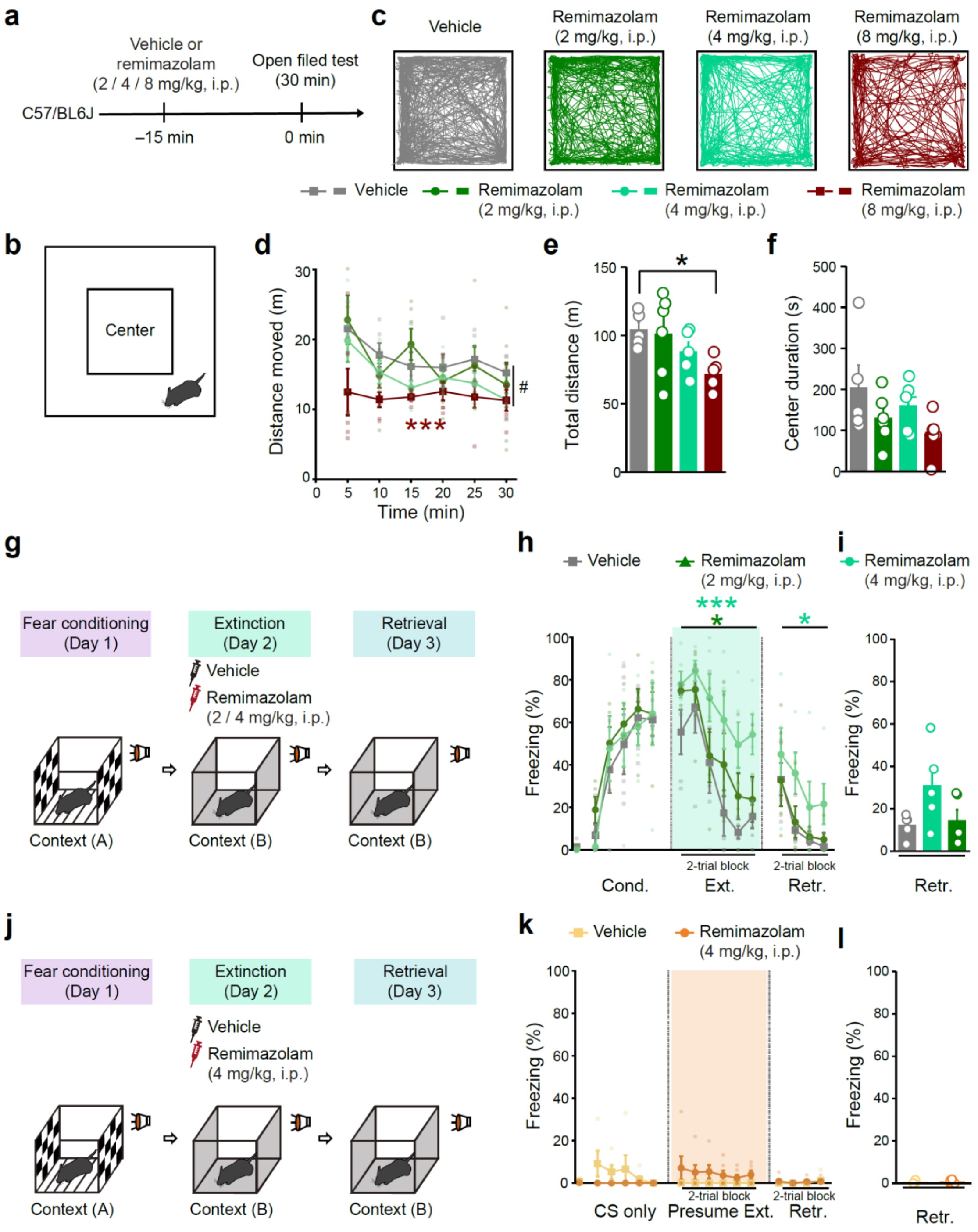

Supplementary Fig. 1. Behavioral effects of remimazolam with different doses. a, **g**, **j**, Experimental design. **b**, Schematic of open field test. **c**, Example traces for mice moving in the open field. **d**, Distance moved in different time bins. Statistics are as follows: two-way repeated measures ANOVA followed by Bonferroni's multiple comparisons test, $F_{(3, 18)} = 3.351$, $^{*}P = 0.0421$; Vehicle vs. Remimazolam (2 mg/kg), $P > 0.9999$; Vehicle vs. Remimazolam (4 mg/kg), $P = 0.1043$; Vehicle vs. Remimazolam

(8 mg/kg), $^{***}P < 0.0001$. Vehicle group, n = 5 mice; Remimazolam (2 mg/kg) group, n = 6 mice; Remimazolam (4 mg/kg) group, n = 5 mice; Remimazolam (8 mg/kg) group, n = 6 mice. **e**, Total distance. Statistics are as follows: one-way ANOVA followed by Bonferroni's multiple comparisons test, $F_{(3, 18)} = 3.351$, $^{*}P = 0.0421$, vehicle vs. Remimazolam 1, $P > 0.9999$, vehicle vs. Remimazolam 2, $P = 0.6234$, vehicle vs. Remimazolam 3, $^{*}P = 0.0371$. **f**, Time spent in the center zone of the open field. Statistics are as follows: one-way ANOVA followed by Bonferroni's multiple comparisons test, $F_{(3, 18)} = 2.084$, $P = 0.1382$; Vehicle vs. Remimazolam (2 mg/kg), $P = 0.3817$; vehicle vs. Remimazolam (4 mg/kg), $P > 0.9999$; vehicle vs. Remimazolam (8 mg/kg), $P = 0.0803$. **h**, **i**, Effects of systemic (i.p.) remimazolam with different doses on fear extinction. Vehicle group, n = 5 mice; Remimazolam (2 mg/kg), n = 5 mice; Remimazolam (4 mg/kg), n = 5 mice. **h**, Time course of freezing responses to the CS. Statistics are as follows: two-way repeated measures ANOVA followed by Bonferroni's multiple comparisons test, Cond.: $F_{(2, 12)} = 0.3294$, $P = 0.7257$; Vehicle vs. Remimazolam (2 mg/kg), $P = 0.8238$; Vehicle vs. Remimazolam (4 mg/kg), $P > 0.9999$; Ext.: $F_{(2, 12)} = 3.916$, $^{*}P = 0.049$; Vehicle vs. Remimazolam (2 mg/kg), $P = 0.3079$; Vehicle vs. Remimazolam (4 mg/kg), $^{***}P < 0.0001$; Retr.: $F_{(2, 12)} = 2.913$, $P = 0.0931$; Vehicle vs. Remimazolam (2 mg/kg), $P = 0.9739$; Vehicle vs. Remimazolam (4 mg/kg), $^{*}P = 0.0248$. **i**, Freezing responses during extinction retrieval. Statistics are as follows: one-way ANOVA, $F_{(2, 12)} = 2.913$, $P = 0.0931$; Vehicle vs. Remimazolam (2 mg/kg), $P > 0.9999$; Vehicle vs. Remimazolam (4 mg/kg), $P = 0.1431$. **k**, **l**, Effects of systemic (i.p.) remimazolam (4 mg/kg) on freezing levels in mice subjected to similar conditioning and extinction protocols but without foot shocks (CS only). Vehicle group, n = 5 mice; Remimazolam group, n = 6 mice. Statistics are as follows: **k**, two-way repeated measures ANOVA, Cond.: $F_{(1, 9)} = 2.875$, $P = 0.1242$; Ext.: $F_{(1, 9)} = 2.443$, $P = 0.1525$; Retr.: $F_{(1, 9)} = 0.2072$, $P = 0.6597$. **l**, two-tailed unpaired Student's *t*-test, $t_{(9)} = 0.4552$, $P = 0.6597$. Data are presented as mean ± SEM.

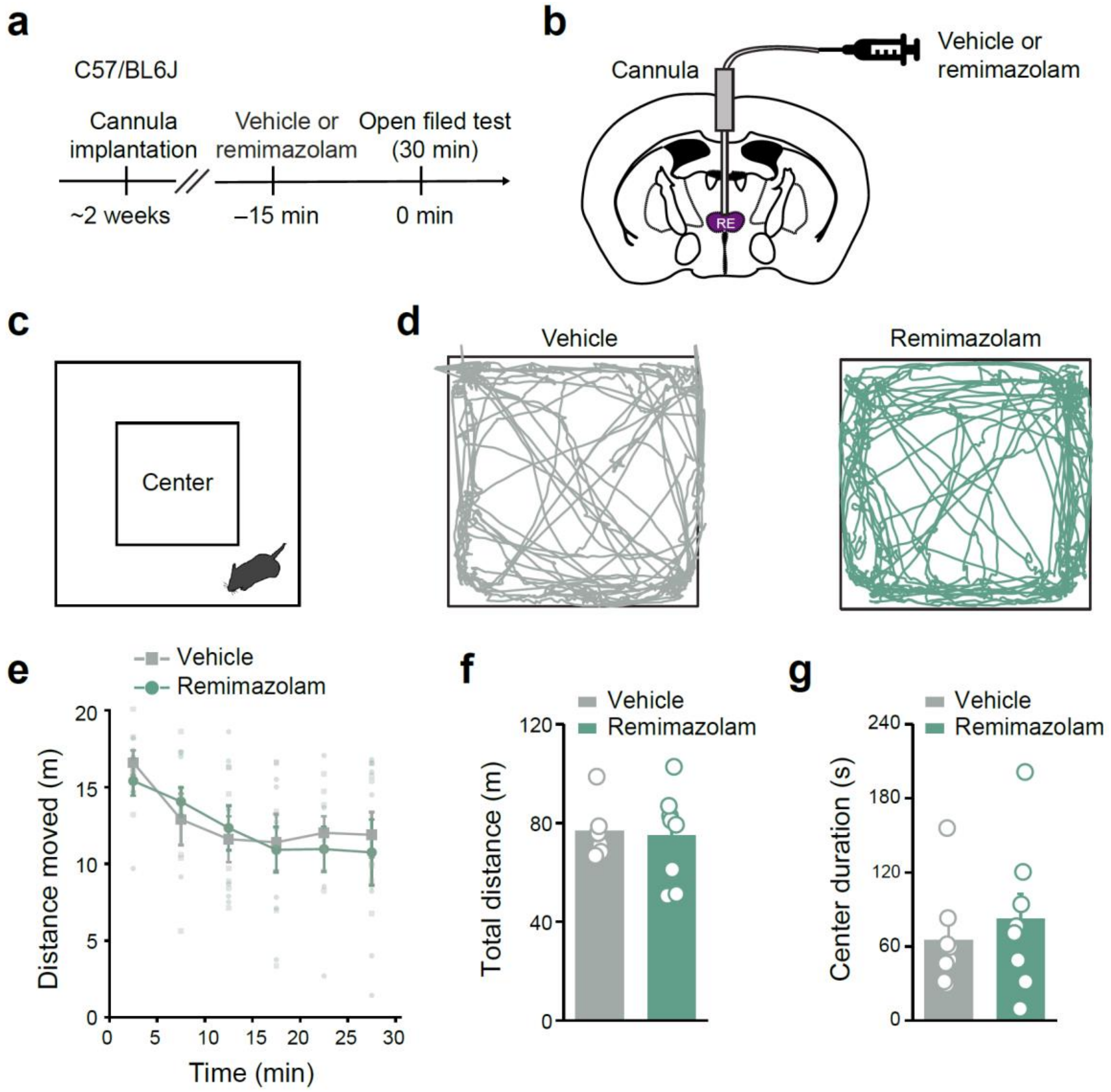

Supplementary Fig. 2. Effects of RE-specific infusion of remimazolam on locomotor activity and basal anxiety. **a**, Experimental design. **b**, Schematics of cannula implantation in RE for drug delivery. **c**, Schematic of the open field test. **d**, Example traces of mice moving in the open field with the application of vehicle (*Left*) or Remimazolam (*Right*). **e**, Distance moved in the open field at different time bins. Statistics are as follows: two-way repeated measures ANOVA, $F_{(1, 13)} = 0.06311$, $P = 0.8056$. **f**, Total distance moved in the open field. Statistics are as follows: two-tailed unpaired Student's *t*-test, $t_{(13)} = 0.2512$, $P = 0.8056$. Vehicle group, n = 7 mice, Remimazolam group, n = 8 mice. **g**, Time spent in the center of the open field. Statistics are as follows: two-tailed unpaired Student's *t*-test, $t_{(14)} = 0.6680$, $P = 0.5150$. Data are presented as mean ± SEM.

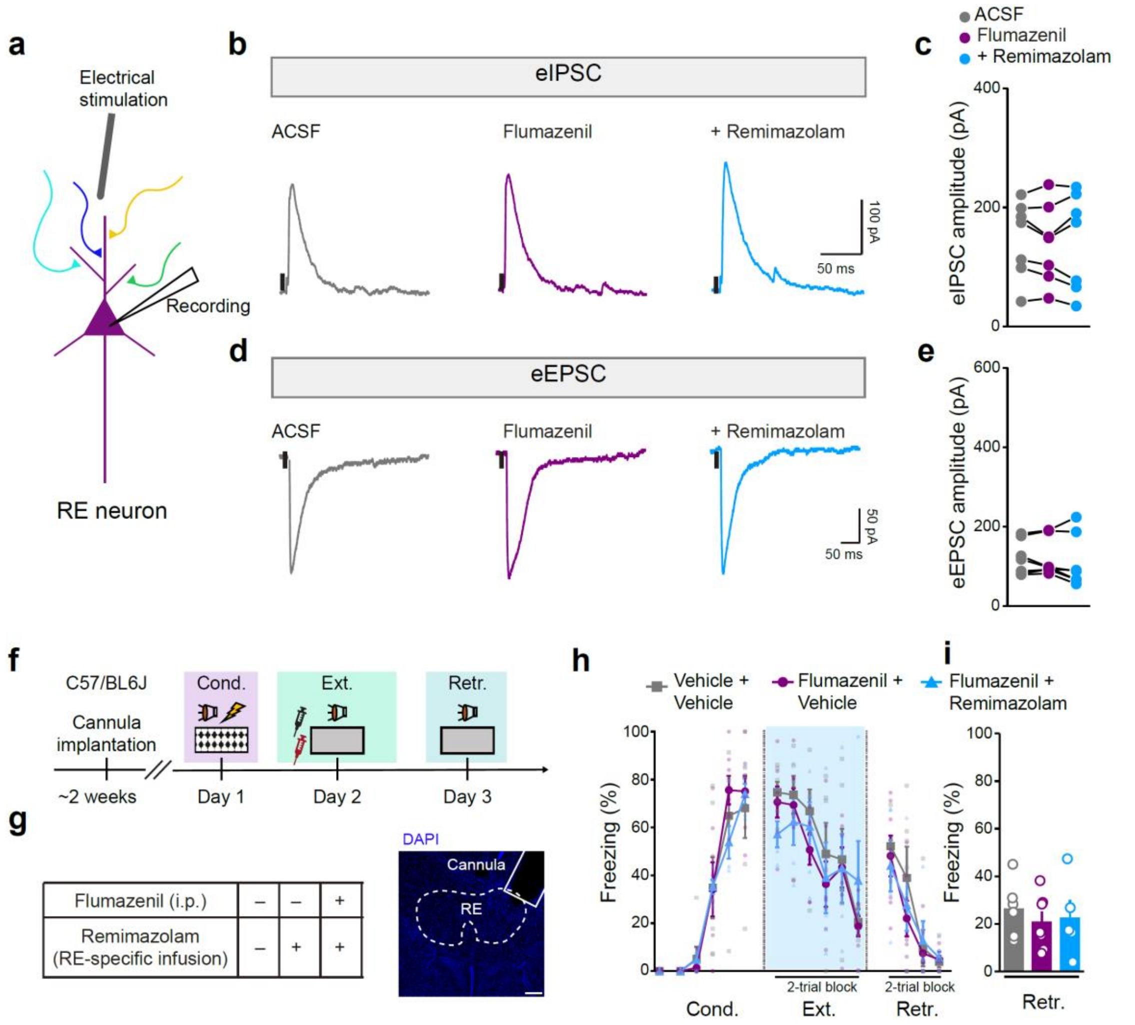

Supplementary Fig. 3. Flumazenil abolishes the effects of remimazolam on RE synaptic transmission and fear extinction behaviors. a–**e**, Effects of flumazenil or flumazenil plus remimazolam on synaptic responses of RE neurons. **a,** Schematic diagram of whole-cell patch clamp recording. **b**, **d**, Example traces of eIPSCs (**b**, holding = 0 mV) and eEPSCs (**d**, holding = -70 mV). **c**, **e**, Statistics are as follows: one-way ANOVA, **c**, $F_{(1.754, 10.52)} = 0.5075$, $P = 0.5927$; **e**, $F_{(1.247, 7.480)} = 0.7782$, $P = 0.4339$. n = 7 cells. **f**–**i**, Effects of flumazenil or flumazenil plus remimazolam on fear extinction. **f**, Experimental design. **g**, *Left*, Schematics of drug delivery. *Right*, Representative image of implantation sites in RE. Scale bar, 200 µm. **h**, **i**, Vehicle + Vehicle group, n = 6 mice; Flumazenil + Vehicle group, n = 7 mice; Flumazenil + Remimazolam group, n = 5 mice. **h**, Time course of freezing responses to the CS. Statistics are as follows: two-way repeated measures ANOVA followed by Bonferroni's multiple comparisons test, Cond.: $F_{(2, 15)} = 0.1952$, $P = 0.8248$; Vehicle + Vehicle vs. Flumazenil + Vehicle, $P > 0.9999$; Vehicle + Vehicle vs. Flumazenil + Remimazolam, $P > 0.9999$; Flumazenil

+ Vehicle vs. Flumazenil + Remimazolam, $P > 0.9999$; Ext.: $F_{(2, 15)} = 0.3010$, $P = 0.7445$; Vehicle + Vehicle vs. Flumazenil + Vehicle, $P = 0.7887$; Vehicle + Vehicle vs. Flumazenil + Remimazolam, $P > 0.9999$; Flumazenil + Vehicle vs. Flumazenil + Remimazolam , $P > 0.9999$; Retr.: $F_{(2, 15)} = 0.3073$, $P = 0.7399$; Vehicle + Vehicle vs. Flumazenil + Vehicle, $P = 0.8197$; Vehicle + Vehicle vs. Flumazenil + Remimazolam, $P = 0.9465$; Flumazenil + Vehicle vs. Flumazenil + Remimazolam, $P = 0.9911$. **i**, Freezing responses during extinction retrieval. Statistics are as follows: one-way ANOVA followed by Bonferroni's multiple comparisons test, $F_{(2, 15)} = 0.3073$, $P = 0.7399$, Vehicle + Vehicle vs. Flumazenil + Vehicle, $P = 0.7887$; Vehicle + Vehicle vs. flumazenil + Remimazolam, $P > 0.9999$; Flumazenil + Vehicle vs. Flumazenil + Remimazolam, $P > 0.9999$. Data are presented as mean ± SEM.

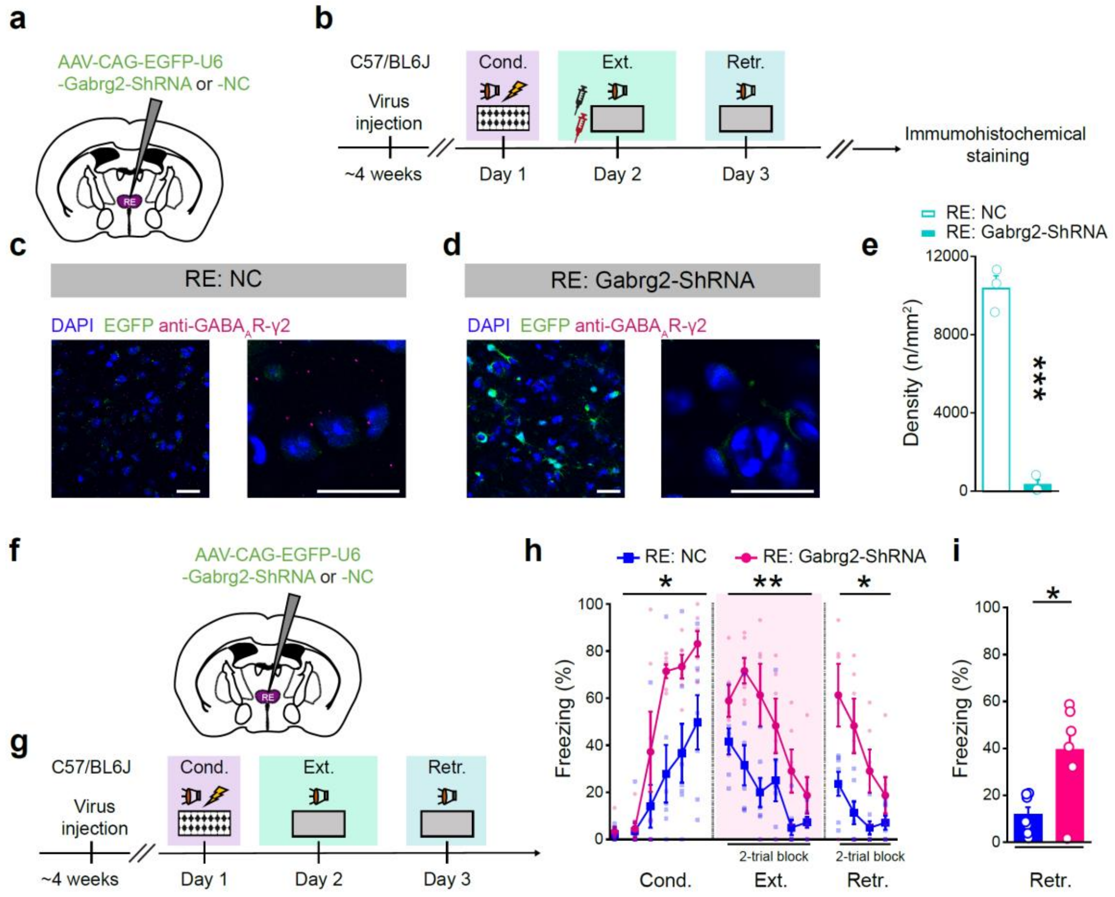

Supplementary Fig. 4. Verification of Gabrg2 knock down in RE and related behavioral effects. a, **f**, Schematics of AAV injections. **b**, **g**, Experimental design. **c**, **d**, Representative images of immunostaining of Gabrg2 in RE-NC (**c**) or RE-Gabrg2 group (**d**). Scale bars, 20 µm. **e,** Quantification of Gabrg2 puncta in RE-NC and RE-Gabrg2 group. n = 3 mice per group. Statistics are as follows: two-tailed unpaired Student's *t*-test, $t_{(4)}$ = 14.61, $^{***}P$ = 0.0001. **h**, **i**, Behavioral effects of Gabrg2 knock down in RE. NC group, n = 7 mice; Gabrg2-ShRNA group, n = 6 mice. **h**, Time course of freezing responses to the CS. Statistics are as follows: two-way repeated measures ANOVA, Cond.: $F_{(1, 11)}$ = 7.795, $^{*}P$ = 0.0175; Ext.: $F_{(1, 11)}$ = 10.97, $^{**}P$ = 0.0069; Retr.: $F_{(1, 11)}$ = 7.399, $^{*}P$ = 0.0199. **i**, Freezing responses during extinction retrieval. Statistics are as follows: two-tailed unpaired Student's *t*-test, $t_{(9)}$ = 2.560, $^{*}P$ = 0.0307. Data are presented as mean ± SEM.

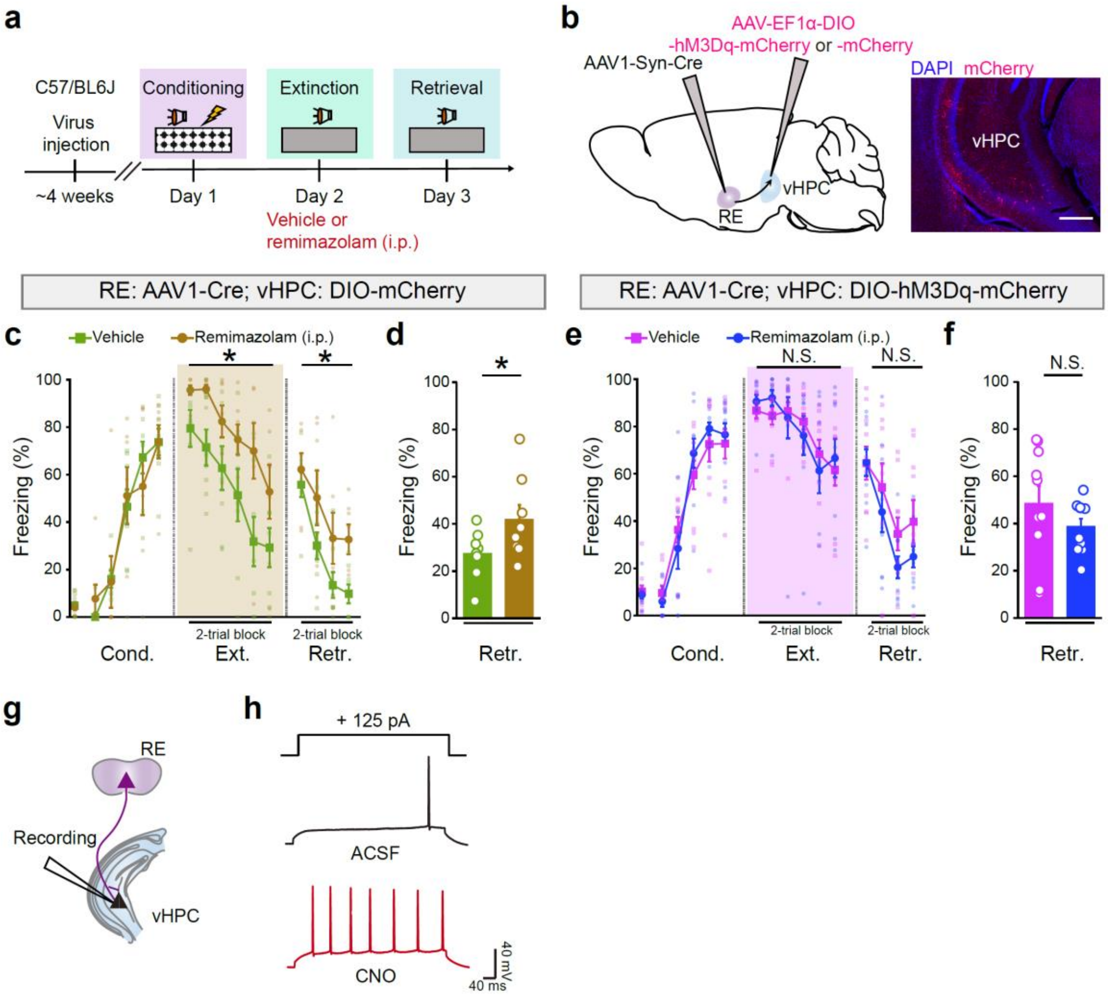

Supplementary Fig. 5. Effects of chemogenetic activating RE-vHPC projectors on the remimazolam responsiveness to fear extinction. **a**, Experimental design. **b**, Schematic of AAV injections (*Left*) and representative images of mCherry expression (red) in vHPC (*Right*). Scale bar, 200 μm. **c**–**f**, Effects of remimazolam (i.p.) in mice with mCherry- (**c**, **d**) and hM3Dq-expressing (**e**, **f**) RE-vHPC projectors on fear extinction. **c**, **d**, Vehicle group, n = 8 mice; remimazolam group, n = 10 mice. **e**, **f**, Vehicle group, n = 8 mice; remimazolam group, n = 7 mice. **c**, **e**, Time course of freezing responses to the CS. Statistics are as follows: two-way repeated measures ANOVA, **c**, Cond.: $F_{(1, 13)} = 0.0006584$, $P = 0.9799$; Ext.: $F_{(1, 13)} = 6.143$, $^{*}P = 0.0277$; Retr.: $F_{(1, 13)} = 5.800$, $^{*}P = 0.0316$; **e**, Cond.: $F_{(1, 18)} = 0.07660$, $P = 0.7851$; Ext.: $F_{(1, 18)} = 0.0004152$, $P = 0.9840$; Retr.: $F_{(1, 18)} = 0.0004152$, $P = 0.9840$. **d**, **f**, Freezing responses during extinction retrieval. Statistics are as follows: two-tailed unpaired Student's *t*-test, **d**, $t_{(13)} = 2.408$, $^{*}P = 0.0316$, (**f**) $t_{(18)} = 1.167$, $P = 0.2585$. **g**, Schematic of whole-cell patch clamp recording. **h**, Example traces of stepping current injection-induced action potentials before (*Upper*) and after (*Lower*) bath application of CNO

(10 μM). Data are presented as mean ± SEM.

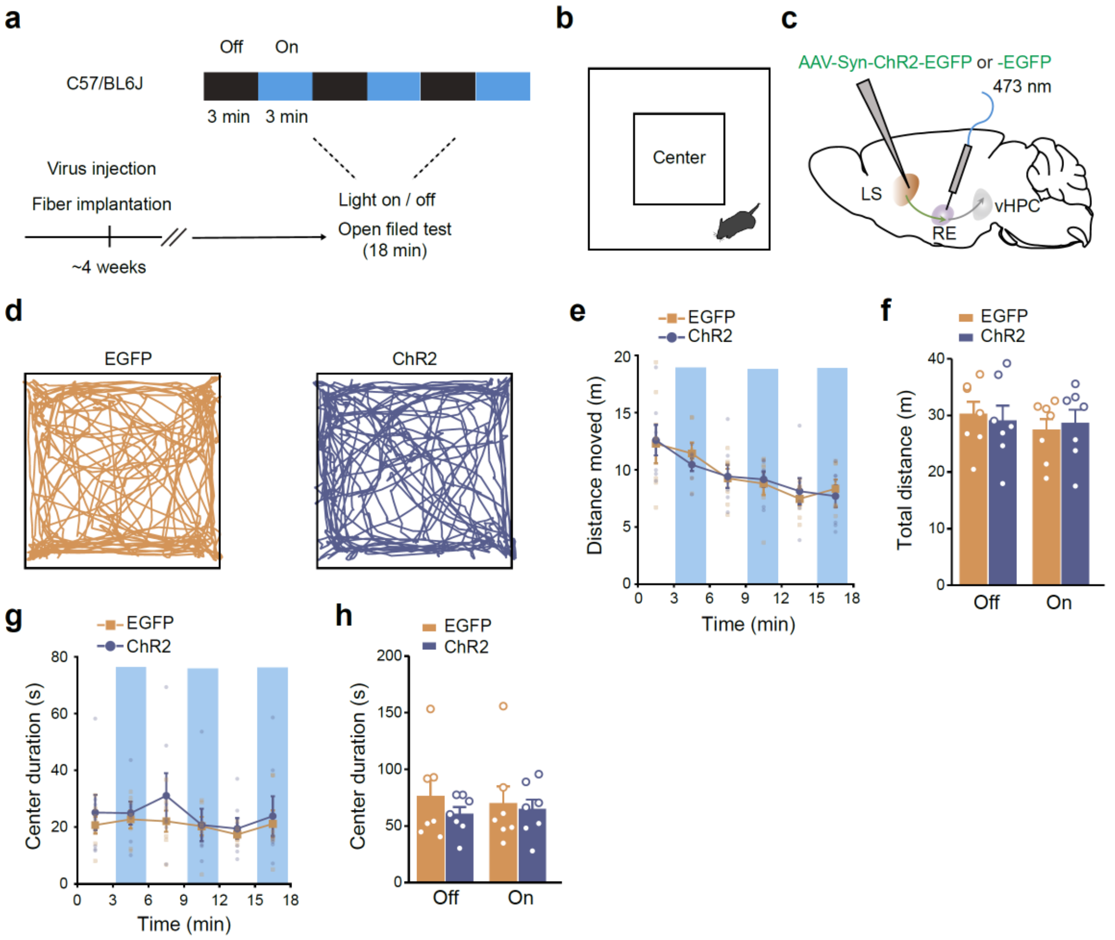

Supplementary Fig. 6. Effects of optogenetic activation of LS → RE projections on locomotor activity and basal anxiety. **a**, Experimental design. **b**, Schematic of the open field test. **c**, Schematic of AAV injections. **d**, Example traces for mice moving in the open field. **e**, Distance moved in the open field in different time bins. Statistics are as follows: two-way repeated measures ANOVA, $F_{(1, 12)}$ = 0.002898, P = 0.9580. mCherry group, n = 7 mice; ChR2 group, n = 7 mice. **f**, Total distances traveled in the open field for both light-on and light-off periods. Statistics are as follows: two-tailed unpaired Student's *t*-test, light off, mCherry vs. ChR2, $t_{(12)}$ = 0.3193, P = 0.7550; light on, mCherry vs. ChR2, $t_{(12)}$ = 0.3940, P = 0.7005. **g**, Time spent in the center of the open field in different time bins. Statistics are as follows: two-way repeated measures ANOVA, $F_{(1, 12)}$ = 0.3887, P = 0.5447. **h**, Time spent in the center of the open field for both light-on and light-off periods. Statistics are as follows: two-tailed unpaired Student's *t*-test, light off, mCherry vs. ChR2, $t_{(12)}$ = 0.9329, P = 0.3693; light on, mCherry vs. ChR2, $t_{(12)}$ = 0.2971, P = 0.7715. Data are presented as mean ± SEM.

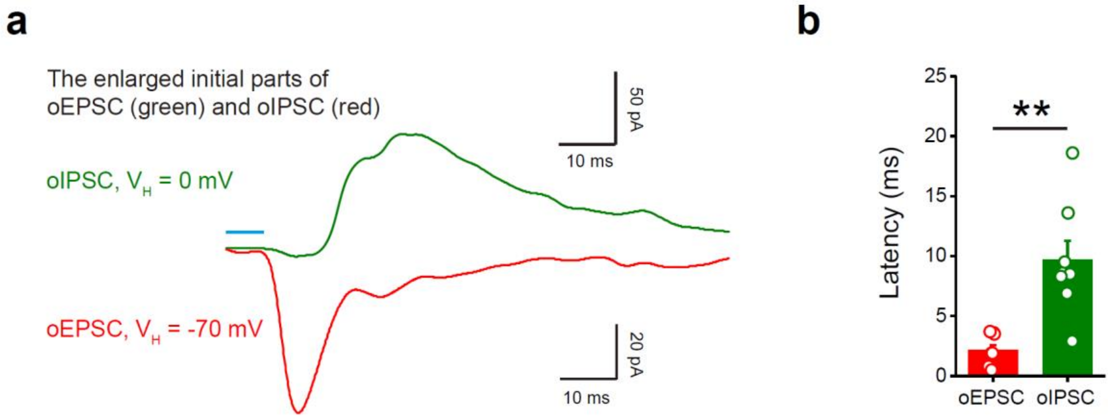

Supplementary Fig. 7. Electrophysiological characterization of synaptic projections from the vHPC to the LS. a, The expanded initial segments of oEPSC (red) and oIPSC (green) in an example LS neuron. Blue bar indicates the duration of light stimulation (5 ms). **b**, Quantification of latency of synaptic connectivity on vHPC-LS projections, n = 8 neurons. Statistics are as follows: two-tailed paired Student's *t*-test, $t_{(7)} = 5.236$, $^{**}P = 0.0012$. Data are presented as mean ± SEM.
