# Supplementary material for: An ultra-short-acting benzodiazepine in thalamic nucleus reuniens undermines fear extinction via intermediation of hippocamposeptal circuits
Source: Commun Biol. 2024 Jun 14;7:728. doi: 10.1038/s42003-024-06417-w (PMC11178775; doi:10.1038/s42003-024-06417-w)
Supplement: Supplementary file 5 — Reporting summary [file 42003_2024_6417_MOESM5_ESM.pdf]

nature portfolio

Corresponding author(s): Wei-Guang Li, Ma-Zhong Zhang, Yan Luo

Last updated by author(s): May 22, 2024

Reporting Summary

Nature Portfolio wishes to improve the reproducibility of the work that we publish. This form provides structure for consistency and transparency in reporting. For further information on Nature Portfolio policies, see our Editorial Policies and the Editorial Policy Checklist.

Statistics

For all statistical analyses, confirm that the following items are present in the figure legend, table legend, main text, or Methods section.

n/a	Confirmed	
☐	☒	The exact sample size (*n*) for each experimental group/condition, given as a discrete number and unit of measurement
☐	☒	A statement on whether measurements were taken from distinct samples or whether the same sample was measured repeatedly
☐	☒	The statistical test(s) used AND whether they are one- or two-sided<br>*Only common tests should be described solely by name; describe more complex techniques in the Methods section.*
☐	☒	A description of all covariates tested
☐	☒	A description of any assumptions or corrections, such as tests of normality and adjustment for multiple comparisons
☐	☒	A full description of the statistical parameters including central tendency (e.g. means) or other basic estimates (e.g. regression coefficient) AND variation (e.g. standard deviation) or associated estimates of uncertainty (e.g. confidence intervals)
☐	☒	For null hypothesis testing, the test statistic (e.g. *F*, *t*, *r*) with confidence intervals, effect sizes, degrees of freedom and *P* value noted<br>*Give P values as exact values whenever suitable.*
☒	☐	For Bayesian analysis, information on the choice of priors and Markov chain Monte Carlo settings
☒	☐	For hierarchical and complex designs, identification of the appropriate level for tests and full reporting of outcomes
☒	☐	Estimates of effect sizes (e.g. Cohen's *d*, Pearson's *r*), indicating how they were calculated

Our web collection on statistics for biologists contains articles on many of the points above.

Software and code

Policy information about availability of computer code

Data collection	pCLAMP 10.5 (Molecular Devices, USA)<br>Olympus VS200 (Olympus, Japan)<br>ANY-maze software 7.3 (UGO BASILE srl, Italy)
Data analysis	GraphPad Prism (version 8.0.2, GraphPad Software, Inc., USA)<br>pCLAMP 10.5 (Molecular Devices, USA)<br>ImageJ (version 1.8.0, NIH, USA)<br>OlyVIA 4.1.1 (Olympus, Japan)<br>ANY-maze software 7.3 (UGO BASILE srl, Italy)

For manuscripts utilizing custom algorithms or software that are central to the research but not yet described in published literature, software must be made available to editors and reviewers. We strongly encourage code deposition in a community repository (e.g. GitHub). See the Nature Portfolio guidelines for submitting code & software for further information.

Data

Policy information about availability of data

All manuscripts must include a data availability statement. This statement should provide the following information, where applicable:

- Accession codes, unique identifiers, or web links for publicly available datasets
- A description of any restrictions on data availability
- For clinical datasets or third party data, please ensure that the statement adheres to our policy

All data supporting the findings of this study are available within the paper and its Supplementary Information. Any additional information required to reanalyze data reported in this paper is available from the lead contact upon request.

Research involving human participants, their data, or biological material

Policy information about studies with human participants or human data. See also policy information about sex, gender (identity/presentation), and sexual orientation and race, ethnicity and racism.

Reporting on sex and gender	No human participants or human data.
Reporting on race, ethnicity, or other socially relevant groupings	No human participants or human data.
Population characteristics	No human participants or human data.
Recruitment	No human participants or human data.
Ethics oversight	No human participants or human data.

Note that full information on the approval of the study protocol must also be provided in the manuscript.

Field-specific reporting

Please select the one below that is the best fit for your research. If you are not sure, read the appropriate sections before making your selection.

☒ Life sciences ☐ Behavioural & social sciences ☐ Ecological, evolutionary & environmental sciences

For a reference copy of the document with all sections, see nature.com/documents/nr-reporting-summary-flat.pdf

Life sciences study design

All studies must disclose on these points even when the disclosure is negative.

Sample size	We sought analogous studies in existing literature and selected sample sizes employed in those investigations. Additionally, a pilot study was undertaken prior to formal experiments, revealing that the standard deviation in our designs closely aligned with the published data.
Data exclusions	No data were excluded from the analyses.
Replication	Yes; all attempts at replication were successful.
Randomization	Randomization was used to assign all experimental groups without bias based on any covariates.
Blinding	All experiments were conducted using a double-blind approach for data collection and statistical analysis

Reporting for specific materials, systems and methods

We require information from authors about some types of materials, experimental systems and methods used in many studies. Here, indicate whether each material, system or method listed is relevant to your study. If you are not sure if a list item applies to your research, read the appropriate section before selecting a response.

Materials & experimental systems

n/a	Involved in the study
☐	☒ Antibodies
☒	☐ Eukaryotic cell lines
☒	☐ Palaeontology and archaeology
☐	☒ Animals and other organisms
☒	☐ Clinical data
☒	☐ Dual use research of concern
☒	☐ Plants

Methods

n/a	Involved in the study
☒	☐ ChIP-seq
☒	☐ Flow cytometry
☒	☐ MRI-based neuroimaging

Antibodies

Antibodies used

Primary antibodies:
Rabbit anti-c-fos primary antibody (Cell Signaling Technology, catalog no. 2250, USA)
Secondary antibodies:
Donkey anti-Rabbit IgG (H+L) Highly Cross-Adsorbed Secondary Antibody, Alexa Fluor™ 555 (Thermo Fisher Scientific, Cat#A31572)

Validation

All the antibodies used in the study were commercially available and the more information is available in the manufacturer website.

Animals and other research organisms

Policy information about studies involving animals; ARRIVE guidelines recommended for reporting animal research, and Sex and Gender in Research

Laboratory animals

The experimental procedures involving animals were conducted in strict compliance with the ethical guidelines set forth by the Animal Ethics Committee of Shanghai Jiao Tong University School of Medicine and the Institutional Animal Care and Use Committee (Department of Laboratory Animal Science, Shanghai Jiao Tong University School of Medicine).The studies were carried out on conscious, unconfined male mice aged between 8 to 12 weeks, all from the C57BL/6J strain. These mice were raised in a specific pathogen-free environment within the laboratory animal facilities, maintained under optimal conditions featuring temperatures ranging from 21 to 23 °C, humidity levels between 40 to 60%, and a consistent 12-hour light/dark cycle. The mice had continuous access to standard rodent food and water. Adult male mice within the age bracket of 6 to 12 weeks were selected for a variety of experimental procedures. All experimental interventions were conducted during the daylight period of the light/dark cycle, adhering strictly to the institutional guidelines.

Wild animals

The study did not involve wild animals.

Reporting on sex

Our experimental protocol incorporates behavioral studies. To mitigate potential variability introduced by hormonal fluctuations associated with the estrous cycle in female mice, we deliberately opted for an exclusive utilization of male subjects. This strategic choice enhances the stability and reliability of our experimental outcomes by minimizing confounding factors related to hormonal dynamics.

Field-collected samples

The study did not involve samples collected from the field.

Ethics oversight

All of the animal procedures were approved by the Animal Ethics Committee of Shanghai Jiao Tong University School of Medicine by the Institutional Animal Care and Use Committee (Department of Laboratory Animal Science, Shanghai Jiao Tong University School pf Medicine; Policy Number DLAS-MP-ANIM. 01-05).

Note that full information on the approval of the study protocol must also be provided in the manuscript.

Plants

Seed stocks

Report on the source of all seed stocks or other plant material used. If applicable, state the seed stock centre and catalogue number. If plant specimens were collected from the field, describe the collection location, date and sampling procedures.

Novel plant genotypes

Describe the methods by which all novel plant genotypes were produced. This includes those generated by transgenic approaches, gene editing, chemical/radiation-based mutagenesis and hybridization. For transgenic lines, describe the transformation method, the number of independent lines analyzed and the generation upon which experiments were performed. For gene-edited lines, describe the editor used, the endogenous sequence targeted for editing, the targeting guide RNA sequence (if applicable) and how the editor was applied.

Authentication

Describe any authentication procedures for each seed stock used or novel genotype generated. Describe any experiments used to assess the effect of a mutation and, where applicable, how potential secondary effects (e.g. second site T-DNA insertions, mosiacism, off-target gene editing) were examined.
